# Clinical significance of baseline Pan-Immune-Inflammation Value and its dynamics in metastatic colorectal cancer patients under first-line chemotherapy

**DOI:** 10.1038/s41598-022-10884-8

**Published:** 2022-04-27

**Authors:** Martín Pérez-Martelo, Alejandro González-García, Yolanda Vidal-Ínsua, Cristina Blanco-Freire, Elena María Brozos-Vázquez, Ihab Abdulkader-Nallib, Javier Álvarez-Fernández, Héctor Lázare-Iglesias, Carolina García-Martínez, Yoel Z. Betancor, María Sánchez-Ares, Jose M. C. Tubío, Francisca Vázquez-Rivera, Sonia Candamio-Folgar, Rafael López-López, Juan Ruiz-Bañobre

**Affiliations:** 1 Medical Oncology Department, University Clinical Hospital of Santiago de Compostela, University of Santiago de Compostela (USC), Travesía da Choupana S/N, 15706 Santiago de Compostela, Spain; 2 Translational Medical Oncology Group (Oncomet), Health Research Institute of Santiago de Compostela (IDIS), University Clinical Hospital of Santiago de Compostela, University of Santiago de Compostela (USC), 15706 Santiago de Compostela, Spain; 3 Pathology Department, University Clinical Hospital of Santiago de Compostela, University of Santiago de Compostela (USC), 15706 Santiago de Compostela, Spain; 4 Medical Oncology Department, Lucus Augusti University Hospital, 27003 Lugo, Spain; 5 Genomes and Disease, Centre for Research in Molecular Medicine and Chronic Diseases (CiMUS), University of Santiago de Compostela (USC), 15706 Santiago de Compostela, Spain; 6 Centro de Investigación Biomédica en Red Cáncer (CIBERONC), 28029 Madrid, Spain

**Keywords:** Prognostic markers, Colorectal cancer

## Abstract

Pan-Immune-Inflammation Value (PIV) has been recently proposed as a new blood-based prognostic biomarker in metastatic colorectal cancer (mCRC). Herein we aimed to validate its prognostic significance and to evaluate its utility for disease monitoring in patients with mCRC receiving first-line chemotherapy. We conducted a single-centre retrospective study involving 130 previously untreated mCRC patients under first-line standard chemotherapy in a real-world scenario. PIV was calculated as *(neutrophil count* × *platelet count* × *monocyte count)/lymphocyte count* at three different time-points: baseline, week 4 after therapy initiation, and at disease progression. We analyzed the influence of baseline PIV on overall survival (OS), progression-free survival (PFS), disease control rate (DCR), and overall response rate (ORR). We also explored the utility of PIV dynamics for disease monitoring. Baseline PIV high was significantly associated with worse OS in univariate [hazard ratio (HR) = 2.10, 95% CI, 1.41–3.15; *p* = 0.000299] and multivariate (HR = 1.82, 95% CI, 1.15–2.90; *p* = 0.011) analyses. Baseline PIV was also associated with worse PFS in univariate (HR = 2.04, 95% CI, 1.40–2.97; *p* = 0.000187) and multivariate (HR = 1.56, 95% CI, 1.05–2.31; *p* = 0.026) analyses. Baseline PIV was not correlated either with DCR or ORR. Regarding PIV dynamics, there was a statistically significant increase from week 4 to disease progression (*p* = 0.0003), which was at the expense of cases with disease control as best response (*p* < 0.0001). In conclusion, this study validates the prognostic significance of baseline PIV in patients with mCRC receiving first-line standard chemotherapy in a real-world scenario. Moreover, it suggests the potential utility of PIV monitoring to anticipate the disease progression among those patients who achieve initial disease control.

## Introduction

Chemotherapy has been classically the central treatment of patients with metastatic colorectal cancer (mCRC), and fortunately nowadays there are many types of agents approved in this particular setting: fluoropyrimidines, irinotecan, oxaliplatin, and trifluridine/tipiracil^1^. In addition, in the last years, molecularly-targeted therapies against the epidermal growth factor receptor (EGFR), angiogenic factors, and more recently, V-raf murine sarcoma viral oncogene homolog B1 (BRAF), and programmed cell death 1 (PD-1) have been approved for being used in the clinical practice^2–4^. Fully aware of the importance of prognostic and predictive biomarkers, the medical and scientific community has driven multiple initiatives for the development and implementation of these essential tools in a clinical context where different treatment options with a comparable level of evidence are available^2^.

In the era of precision oncology, blood-based biomarkers emerge as a promising alternative for their tissue counterparts. Several groups have described the prognostic value of different inflammatory markers, such as neutrophil to lymphocyte ratio (NLR)^5^, derived neutrophil to lymphocyte ratio (dNLR)^6^, lymphocyte to monocyte ratio^7^, platelet-lymphocyte ratio^8^, systemic immune-inflammation index (SII)^9^, and more recently, the Pan-Immune-Inflammation Value (PIV)^10^. In 2020 Fucà et al.^10^ have described a novel blood-based biomarker, the PIV, which integrating most of the immune-inflammatory peripheral blood cells with proved prognostic relevance in the advanced setting, was able to stratify mCRC patients under first-line chemotherapy according to survival outcomes^10^. In addition they also demonstrated a better performance of PIV compared with some previously well-described inflammatory-related markers such as SII, NLR, and monocyte and platelet counts^10^. On similar lines, in 2021 Corti et al.^11^ evaluated and confirmed the prognostic significance of PIV in a cohort of 163 DNA MMR-deficient/Microsatellite instability-high (MSI-H) mCRC receiving either PD-1/programmed death-ligand 1 (PD-L1) antibodies alone or in combination with Cytotoxic T-Lymphocyte Antigen 4 (CTLA-4) blockade.

Taking into consideration the importance of validating biomarkers in the real-world clinical scenario, we conducted this retrospective study in order to validate the prognostic relevance of PIV and explore the utility of its dynamics for disease monitoring in mCRC patients receiving first-line standard chemotherapy.

## Results

### Patient population

Between October 23, 2015 and January 25, 2018, 130 patients were enrolled. Baseline patient and disease characteristics are summarized in Supplementary Table 1. The median age was 68.8 years (range, 26–88). Twenty-six percent (n = 34) of patients were female and 74% (n = 96) male. All the patients were TNM stage IV at chemotherapy initiation; 78% (n = 101) had metastatic disease in liver, 49% (n = 64) in lung, 18% (n = 23) in peritoneum, 33% (n = 43) in lymph nodes, 2% (n = 3) in bone, and 43% (n = 56) in other different locations. The most frequent primary tumor location was left side (78%, n = 102), and overall, primary tumor was resected in 65% (n = 84) of patients. Twelve percent (n = 16) of the patients had ECOG-PS 0, while 66% (n = 86), 18% (n = 24) and 3% (n = 4) had ECOG-PS 1, 2 and 3, respectively. Fifteen percent (n = 20) of patients received adjuvant chemotherapy, while 6% (n = 8) received neoadjuvant chemoradiotherapy, of which 6 (75%) also received adjuvant chemotherapy. Sixty-eight percent (n = 89) of patients received mFOLFOX6, 5% (n = 7) CAPEOX, 17% (n = 22) FOLFIRI, 4% (n = 5) irinotecan, and 5% (n = 7) capecitabine or 5-FU/LV. Baseline complete blood cell counts were available for all included patients. Complete blood cell counts at week 4 from therapy initiation and at disease progression were available for 125 (96%) and 98 (75%) patients, respectively.

Median baseline PIV was 424.05 (range: 30.19–6675.05) in the overall study population, whereas median early PIV change was − 55.10% (range: − 95.74 to 412.15%). Table 1 summarizes the distribution of baseline PIV and early PIV change according to clinicopathological characteristics. Of note, median baseline PIV was significantly higher in patients with ECOG-PS ≥ 2 (*p* < 0.001), synchronous metastases (*p* = 0.024), no primary tumor resection (*p* < 0.001), ≥ 2 metastatic sites at diagnosis (*p* = 0.028), peritoneal (*p* = 0.023) and bone metastases (*p* = 0.036), among those patients who received oxaliplatin-based chemotherapy (*p* = 0.012), and among those patients who did not received adjuvant chemotherapy (*p* = 0.037). Guided by these results, we explored the distribution of different patient and disease characteristics according to chemotherapy regimen (Supplementary Table 3). Interestingly, among patients who received oxaliplatin-based chemotherapy a significantly higher proportion presented synchronous metastases (*p* < 0.001), liver metastases (*p* = 0.015) and lymph node metastases (*p* = 0.039). Additionally, a more pronounced reduction of PIV at week 4 after therapy initiation was observed in patients without primary tumor resection (*p* = 0.021), with synchronous metastases (*p* = 0.025), with peritoneal metastases (*p* = 0.008), and with RAS mutated tumors (*p* = 0.030).

**Table 1:** Baseline PIV and PIV change according to clinicopathological characteristics.

Characteristics	Median baseline PIV (range)	*p* value	Median early PIV change % (range)	*p* value
**Age**		0.074		0.398
≥ Median	366.9 (30.2–6675.1)		− 52.5 (− 90.4 to 412.1)	
< Median	452.3 (49.6–3664.2)		− 55.8 (− 95.7 to 255.1)	
**Sex**		0.488		0.579
Male	423.1 (49.6–6675.1)		− 54.7 (− 95.7 to 412.1)	
Female	526.0 (30.2–3664.2)		− 55.8 (− 85.3 to 155.6)	
**CEA**		***0.005***		0.051
> 5 ng/mL	489.9 (49.6–6675.1)		− 59.1 (− 95.7 to 314.1)	
≤ 5 ng/mL	317.5 (30.2–1530.8)		− 41.2 (− 90.4 to 412.1)	
**BMI**		0.241		0.48
≥ 25	422.5 (30.2–6675.1)		− 54.9 (− 95.7 to 314.1)	
< 25	462.8 (74.1–3664.2)		− 59.1 (− 90.4 to 412.1)	
**ECOG-PS**		**< *0.001***		0.149
≥ 2	799.8 (67.6–3664.2)		− 67.8 (− 94.9 to 155.6)	
0–1	364.3 (30.2–6675.1)		− 53.0 (− 95.7 to 412.1)	
**Chemotherapy**		***0.004***		0.056
Oxaliplatin-based	463.9 (49.6–6675.1)		− 57 (− 95.7 to 314.1)	
Non-oxaliplatin-based	246.7 (30.2–1497.2)		− 41.6 (− 89.2 to 412.2)	
**Synchronous metastases**		***0.024***		***0.025***
Yes	457.2 (49.6–6675.1)		− 57.4 (− 94.9 to 314.1)	
No	318.7 (30.2–1497.2)		− 28.7 (− 95.7 to 412.2)	
**Number of metastatic sites**		***0.028***		0.28
≥ 2	454.9 (30.2–6675.1)		− 56.3 (− 95.7 to 412.2)	
1	337.1 (50.7–2016.7)		− 42.9 (− 90.4 to 95.7)	
**Primary tumor location**		0.353		0.67
Right	523.9 (30.2–6675.1)		− 60.6 (− 90.4 to 412.2)	
Left	398.8 (49.6–3664.2)		− 54.7 (− 95.7 to 314.1)	
**TNM stage at diagnosis**		0.898		0.603
I–II	444.2 (67.6–1050.9)		− 53.8 (− 95.7 to 108.3)	
III–IV	423.5 (30.2–6675.1)		− 55.3 (− 94.4 to 412.2)	
**Liver metastases**		***0.025***		0.127
Yes	462.8 (49.6–6675.1)		− 56.2 (− 95.7 to 412.2)	
No	307.6 (30.2–1064.6)		− 31.9 (− 90.4 to 132.1)	
**Lung metastases**		0.769		0.415
Yes	423.1 (30.2–6675.1)		− 52.5 (− 94.9 to 255.1)	
No	446.3 (50.7–3136.4)		− 57.1 (− 95.7 to 412.2)	
**Peritoneal metastases**		***0.023***		***0.008***
Yes	550.8 (142.8–6675.1)		− 72.9 (− 90.4 to 92.3)	
No	389.8 (30.2–3650.8)		− 52.5 (− 95.7 to 412.2)	
**Lymph node metastases**		0.449		0.586
Yes	422.5 (49.6–6675.1)		− 55.1 (− 95.7 to 412.2)	
No	529.2 (30.2–3014.7)		− 60.5 (− 89.2 to 132.1)	
**Bone metastases**		***0.036***		0.229
Yes	2820.1 (529.2–3136.4)		− 69.7 (− 87.8 to [− 51.3])	
No	422.5 (30.2–6675.1)		− 54.9 (− 95.7 to 412.2)	
**RAS mutation**		0.310		***0.040***
Yes	500.1 (30.2–6675.1)		− 59.7 (− 95.7 to 255.1)	
No	384.3 (50.7–3139.8)		− 44.7 (− 91.3 to 412.2)	
**BRAF mutation**		0.314		0.891
Yes	1234.5 (452.3–2016.7)		− 37.7 (− 79.1 to 3.7)	
No	441.2 (30.2–6675.1)		− 57.0 (− 95.7 to 412.2)	
**MMR deficiency**		0.686		0.585
Yes	1064.1 (209.3–1918.9)		− 33.5 (− 64.7 to [− 2.3])	
No	424.0 (30.2–6675.1)		− 56.0 (− 95.7 to 314.1)	
**Antibody therapy**		0.164		0.936
Yes	397.76 (49.6–3014.7)		− 56.3 (− 95.7 to 412.2)	
No	452.3 (30.2–6675.1)		− 53.8 (− 94.9 to 255.1)	
**Primary tumor resection**		**< *0.001***		***0.021***
Yes	341.1 (30.2–2804.5)		− 44.7 (− 95.7 to 412.2)	
No	669.0 (49.6–6675.1)		− 64.7 (− 94.9 to 155.6)	
**Smoking status**		0.479		0.972
Ever	384.3 (49.6–6675.1)		− 53.0 (− 95.7 to 412.2)	
Never	468.3 (30.2–3664.2)		− 55.6 (− 89.2 to 314.1)	
**Adjuvant chemotherapy**		***0.037***		0.12
Yes	215.9 (30.2–1497.2)		− 26.6 (− 95.7 to 412.2)	
No	449.7 (49.6–6675.1)		− 53.3 (− 94.9 to 314.1)	

*CI* confidence interval, *CEA* carcinoembryonic antigen, *BMI* body mass index, *ECOG-PS* Eastern Cooperative Oncology Group Performance Status, *PIV* Pan-Immune-Inflammation Value.

Bold italics numbers indicate statistically significant values.

When dichotomized PIV based on the previously defined cut-off threshold^10^, fifty-four percent (n = 70) of the patients had a high baseline PIV, while 46% (n = 60) had low baseline PIV. Furthermore, 12% (n = 16) of the patients experienced an early PIV increase, while 84% (n = 109) did not. Remaining 5 patients (4%) could not be classified according to early PIV change because they died prior to week 4. The distribution of different patient and disease characteristics according to baseline PIV and early PIV increase is shown in Supplementary Table 2 and Supplementary Table 4, respectively. While in the baseline PIV high group there was a higher proportion of patients with ECOG-PS ≥ 2 (*p* = 0.001), without primary tumor resection (*p* = 0.003), with peritoneal metastases (*p* = 0.039), and who received oxaliplatin-based chemotherapy (*p* = 0.029), no significant differences in patient and disease characteristics were observed according to early PIV increase.

### Clinical significance of baseline Pan-Immune-Inflammation Value

#### Overall survival

**Figure 1:**
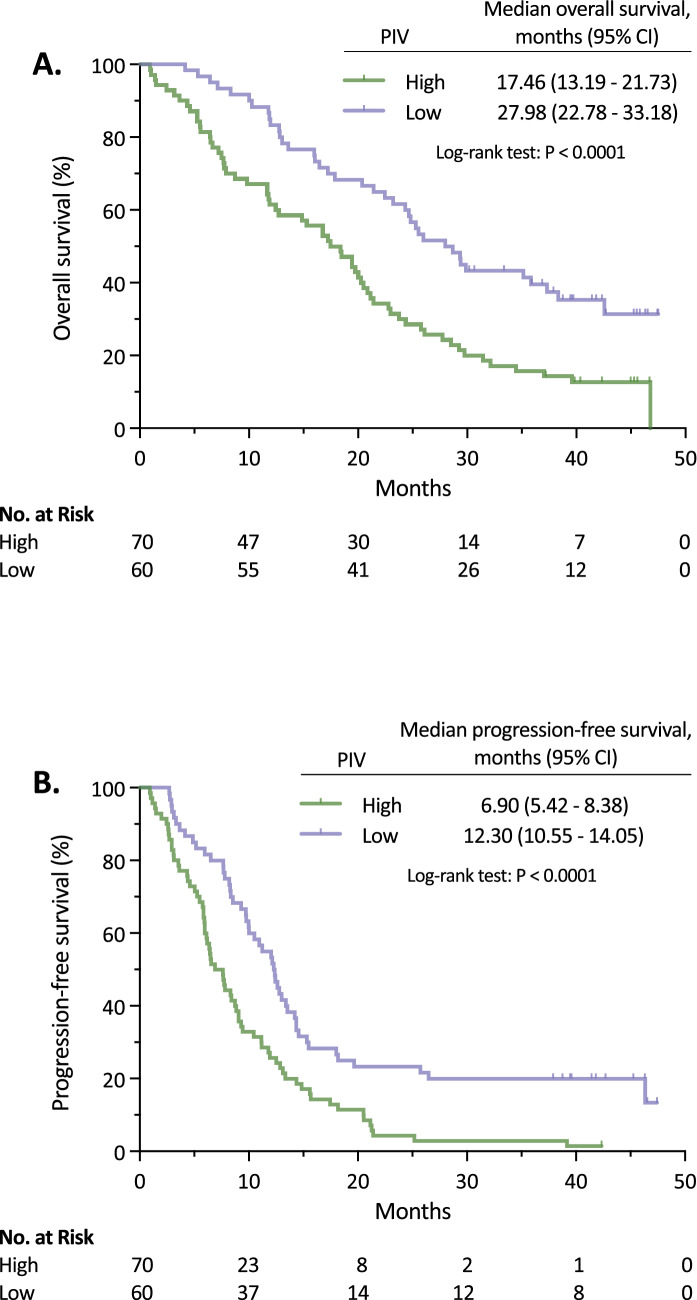
Kaplan–Meier survival estimates according to baseline PIV. (**A**) Overall survival. (**B**) Progression-free survival.

At the time of the data collection, 78% (n = 101) of enrolled patients had died. Median OS was 21.14 months (95% CI, 17.70–24.78), and the 6-, 12-, 24- and 36-month OS rate were 74.62% (95% CI, 60.51–91.02), 65.38% (95% CI, 52.23–80.85), 42.31% (95% CI, 31.87–55.07), and 22.31% (95% CI, 14.94–32.04), respectively (Supplementary Table 5). Median OS for high and low baseline PIV patients was 17.46 months (95% CI, 13.19–21.73), and 27.98 months (95% CI, 22.78–33.18) (*p* < 0.0001), respectively (Fig. 1A). High baseline PIV was significantly associated with worse OS in univariate [hazard ratio (HR) = 2.10, 95% CI, 1.41–3.15; *p* = 0.000299] and multivariate (HR = 1.82, 95% CI, 1.15–2.90; *p* = 0.011) analyses (Table 2). Other baseline variables independently associated with worse OS in multivariate analysis were ECOG-PS ≥ 2 (HR = 2.24, 95% CI, 1.31–3.82; *p* = 0.003), baseline CEA > 5 ng/mL (HR = 1.77, 95% CI, 1.07–2.91; *p* = 0.025), and right primary tumor location (HR = 1.67, 95% CI, 1.02–2.74; *p* = 0.043) (Table 2). On the other hand, primary tumor resection (HR = 0.37, 95% CI, 0.23–0.60; *p* = 0.000052), and presence of lymph node metastases (HR = 0.36, 95% CI, 0.19–0.70; *p* = 0.002) were independently associated with better OS (Table 2).

**Table 2:** Univariate and multivariate Cox regression analyses for overall survival.

Characteristics	Univariate analysis		Multivariate analysis	
	HR (95% CI)	*p* value	HR (95% CI)	*p* value
Age (increment of 1 year)	1.022 (1.002–1.042)	***0.028***	1.015 (0.993–1.037)	0.178
Sex (male vs. female)	1.128 (0.713–1.785)	0.607		
CEA (> 5 ng/mL vs. ≤ 5 ng/mL)	2.117 (1.366–3.280)	***0.001***	1.766 (1.073–2.905)	***0.025***
BMI (≥ 25 vs. < 25)	0.841 (0.540–1.309)	0.444		
ECOG-PS (≥ 2 vs. 0–1)	2.741 (1.745–4.306)	***0.000012***	2.235 (1.306–3.823)	***0.003***
Baseline PIV (high vs. low)	2.105 (1.406–3.152)	***0.000299***	1.823 (1.146–2.898)	***0.011***
Chemotherapy (oxaliplatin-based vs. non-oxaliplatin-based)	0.979 (0.630–1.523)	0.926		
Synchronous metastases (yes vs. no)	1.277 (0.817–1.996)	0.284		
Number of metastatic sites (≥ 2 vs. 1)	2.572 (1.654–3.998)	***0.000027***	1.542 (0.827–2.877)	0.173
Primary tumor location (right vs. left)	1.600 (1.014–2.525)	***0.044***	1.669 (1.016–2.743)	***0.043***
TNM stage at diagnosis (III-IV vs. I–II)	1.239 (0.643–2.385)	0.522		
Liver metastases (yes vs. no)	1.415 (0.874–2.292)	0.158		
Lung metastases (yes vs. no)	1.806 (1.217–2.682)	***0.003***	1.249 (0.683–2.285)	0.469
Peritoneal metastases (yes vs. no)	1.709 (1.053–2.774)	***0.03***	0.934 (0.542–1.609)	0.805
Lymph node metastases (yes vs. no)	0.457 (0.253–0.825)	***0.009***	0.361 (0.188–0.696)	***0.002***
Bone metastases (yes vs. no)	5.016 (1.536–16.384)	***0.008***	1.291 (0.348–4.794)	0.703
RAS mutation (yes vs. no)	1.06 (0.704–1.580)	0.796		
BRAF mutation (yes vs. no)	2.269 (0.549–9.37)	0.258		
MMR deficiency (yes vs. no)	3.029 (0.729–12.59)	0.127		
Antibody therapy (yes vs. no)	0.705 (0.473–1.050)	0.085		
Primary tumor resection (yes vs. no)	0.374 (0.249–0.561)	***0.000002***	0.375 (0.233–0.603)	***0.000052***
Smoking status (ever vs. never)	1.003 (0.679–1.482)	0.989		
Adjuvant chemotherapy (yes vs. no)	0.741 (0.428–1.284)	0.286		

*HR* hazard ratio, *CI* confidence interval, *CEA* carcinoembryonic antigen, *BMI* body mass index, *ECOG-PS* Eastern Cooperative Oncology Group Performance Status, *PIV* Pan-Immune-Inflammation Value.

Bold italics numbers indicate statistically significant values.

As previously described, 9% of patients received a single-agent chemotherapy regimen with either irinotecan, capecitabine or 5-FU/LV. Considering this aspect, we explored the prognostic impact on OS of baseline PIV in this subgroup of patients. As expected, although not statistically significant, high baseline PIV was associated with worse OS (HR = 3.79, 95% CI, 0.88–16.41; *p* = 0.075). On the other hand, when we only considered those patients receiving a combination chemotherapy regimen (either FOLFOX, CAPEOX or FOLFIRI; 91% of the whole study population), high baseline PIV was significantly associated with worse OS in univariate (HR = 2.29, 95% CI, 1.48–3.54; *p* = 0.0002] and multivariate (HR = 1.84, 95% CI, 1.13–3.01; *p* = 0.015) analyses (Supplementary Table 6).

#### Progression-free survival

Median PFS based on 118 (n = 91%) PFS events was 9.30 months (95% CI, 7.62–10.98) (Supplementary Table 5). Median PFS for high and low baseline PIV patients was 6.90 months (95% CI, 5.42–8.38), and 12.30 months (95% CI, 10.55–14.05) (*p* < 0.0001), respectively (Fig. 1B). High baseline PIV was significantly associated with worse PFS in univariate (HR = 2.04, 95% CI, 1.40–2.97; *p* = 0.000187) and multivariate (HR = 1.56, 95% CI, 1.05–2.31; *p* = 0.026) analyses (Table 3). Other baseline variables independently associated with poor PFS in multivariate analysis were ECOG-PS ≥ 2 (HR = 1.78, 95% CI, 1.08–2.91; *p* = 0.022), and ≥ 2 metastatic sites at diagnosis (HR = 2.01, 95% CI, 1.17–3.48; *p* = 0.012) (Table 3). On the other hand, BMI > 25 (HR = 0.52, 95% CI, 0.34–0.81; *p* = 0.004), and primary tumor resection (HR = 0.52, 95% CI, 0.34–0.80; *p* = 0.003) were independently associated with better PFS (Table 3).

**Table 3:** Univariate and multivariate Cox regression analyses for progression-free survival.

Characteristics	Univariate analysis		Multivariate analysis	
	HR (95% CI)	*p* value	HR (95% CI)	*p* value
Age (increment of one year)	1.013 (0.995–1.031)	0.149		
Sex (male vs. female)	0.887 (0.588–1.337)	0.566		
CEA (> 5 ng/mL vs. ≤ 5 ng/mL)	1.616 (1.099–2.376)	0.015	1.180 (0.771–1.806)	0.445
BMI (≥ 25 vs. < 25)	0.620 (0.414–0.929)	***0.021***	0.524 (0.340–0.809)	***0.004***
ECOG-PS (≥ 2 vs. 0–1)	2.124 (1.373–3.286)	***0.001***	1.776 (1.085–2.906)	***0.022***
Baseline PIV (high vs. low)	2.041 (1.404–2.968)	***0.000187***	1.561 (1.054–2.311)	***0.026***
Chemotherapy (oxaliplatin-based vs. non-oxaliplatin-based)	1.097 (0.730–1.647)	0.657		
Synchronous metastases (yes vs. no)	1.498 (0.978–2.296)	0.063		
Number of metastatic sites (≥ 2 vs. 1)	2.474 (1.668–3.672)	***0.000007***	2.014 (1.167–3.475)	***0.012***
Primary tumor location (right vs. left)	1.380 (0.895–2.128)	0.145		
TNM stage at diagnosis (III-IV vs. I–II)	1.472 (0.769–2.818)	0.243		
Liver metastases (yes vs. no)	1.281 (0.828–1.983)	0.267		
Lung metastases (yes vs. no)	2.075 (1.434–3.002)	***0.000107***	1.380 (0.825–2.307)	0.219
Peritoneal metastases (yes vs. no)	1.404 (0.878–2.244)	0.156		
Lymph node metastases (yes vs. no)	0.596 (0.332–1.069)	0.083		
Bone metastases (yes vs. no)	3.895 (1.206–12.580)	***0.023***	2.963 (0.857–10.245)	0.086
RAS mutation (yes vs. no)	0.840 (0.577–1.223)	0.363		
BRAF mutation (yes vs. no)	2.571 (0.619–10.681)	0.194		
MMR deficiency (yes vs. no)	1.803 (0.441–7.368)	0.412		
Antibody therapy (yes vs. no)	0.742 (0.513–1.074)	0.114		
Primary tumor resection (yes vs. no)	0.398 (0.270–0.586)	***0.000003***	0.523 (0.343–0.798)	***0.003***
Smoking status (ever vs. never)	0.809 (0.562–1.164)	0.254		
Adjuvant chemotherapy (yes vs. no)	0.676 (0.404–1.132)	0.136		

*HR* hazard ratio, *CI* confidence interval, *CEA* carcinoembryonic antigen, *BMI* body mass index, *ECOG-PS* Eastern Cooperative Oncology Group Performance Status, *PIV* Pan-Immune-Inflammation Value.

Bold italics numbers indicate statistically significant values.

As we did for OS, we explored the prognostic impact on PFS of baseline PIV in the subgroup of patients receiving a single-agent chemotherapy regimen. Again, although not statistically significant, high baseline PIV was associated with worse PFS (HR = 3.43, 95% CI, 0.78–15.04; *p* = 0.102). On the other hand, when we only considered those patients receiving a combination chemotherapy regimen, high baseline PIV was statistically significantly associated with worse PFS in univariate analysis (HR = 2.11, 95% CI, 1.42–3.147; *p* = 0.0002), with a congruent trend in multivariate analysis (HR = 1.52, 95% CI, 0.98–2.35; *p* = 0.062) (Supplementary Table 7).

#### Disease control and overall response rates

DCR and ORR were 78.46% (95% CI, 63.98–95.25) and 53.08% (95% CI, 41.30–67.17) respectively, including 1 (0.8%) complete response (Supplementary Table 5). Among all the baseline variables examined in multivariate analysis, only one was independently associated with higher DCR [antibody therapy administration; odds ratio (OR) = 5.58, 95% CI, 1.73–18.04; *p* = 0.004] (Supplementary Table 8), and two with lower (TNM stage III–IV at diagnosis; OR = 0.18, 95% CI, 0.04–0.88; *p* = 0.034) and higher (antibody therapy administration; OR = 3.34, 95% CI, 1.58–7.06; *p* = 0.002) ORR, respectively (Supplementary Table 9). Baseline PIV was not correlated either with DCR or ORR.

### Pan-Immune-Inflammation Value dynamics

**Figure 2:**
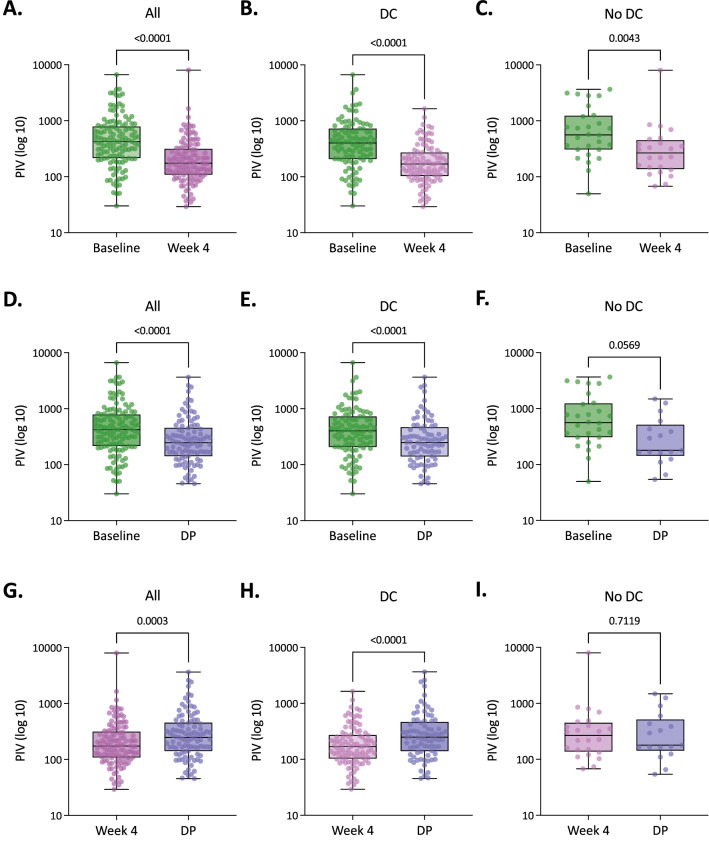
PIV dynamics in patients with metastatic colorectal cancer (mCRC). (**A**) Comparison of PIV at baseline and week 4 from all CRC patients. (**B**) Comparison of PIV at baseline and week 4 from mCRC patients with disease control (DC). (**C**) Comparison of PIV at baseline and week 4 from mCRC patients with no DC. (**D**) Comparison of PIV at baseline and disease progression (DP) from all CRC patients. (**E**) Comparison of PIV at baseline and DP from mCRC patients with DC. (**F**) Comparison of PIV at baseline and DP from mCRC patients with no DC. (**G**) Comparison of PIV at week 4 and DP from all mCRC patients. (**H**) Comparison of PIV at week 4 and DP from mCRC patients with DC. (**I**) Comparison of PIV at week 4 and DP from mCRC patients with no DC. The *y*-axis (log10 scale) represents PIV values. Boxes represent the interquartile range, and the horizontal line across each box indicates the median value. Whiskers indicate variability outside the upper and lower quartiles, and horizontal lines indicate maximum and minimum values. Statistically significant differences were determined using the Wilcoxon matched-pairs signed rank test.

To evaluate PIV utility to monitor disease evolution in the mCRC scenario, we explored its dynamics from baseline to week 4 after therapy initiation and to radiologically-documented disease progression. First, there was a PIV decrease from baseline to week 4 (*p* < 0.0001), which was independent of the disease control status (Fig. 2A–C). Moreover, there was a PIV decrease from baseline to disease progression (*p* < 0.0001), although this was statistically significant only among those patients with disease control as best response (*p* < 0.0001) (Fig. 2D–F). On the other hand, there was an overall PIV increase from week 4 to disease progression (*p* = 0.0003), which was at the expense of cases with disease control as best response (*p* < 0.0001) (Fig. 2G–I). Early PIV increase was not correlated either with survival or disease control (Supplementary Table 10 and Supplementary Table 11). Taken together, these results suggest the utility of PIV monitoring to anticipate the progression of the disease specifically in those mCRC patients who achieve an initial disease control under first-line chemotherapy.

## Discussion

Over the last years, multiple research initiatives have been carried out to discover and implement prognostic and predictive biomarkers to guide the treatment of patients with mCRC. Despite this massive effort, thus far only three markers have translated into routine clinical practice. The first marker are *RAS* gene mutations, which serve as a negative predictive biomarker and correlate with the lack of efficacy to anti-EGFR antibodies cetuximab and panitumumab. The second marker is tumor MSI, which has emerged as a positive predictive marker for anti-PD-1 drugs. Lastly, the third marker is *BRAF V600E* mutation, a positive predictive biomarker for BRAFi-based regimen encorafenib plus cetuximab. Moreover, in addition to all these tumor molecular characteristics, several inflammatory-related markers have been explored in the mCRC setting in last decade. One of these markers, the PIV, have been recently proposed as a new blood-based prognostic biomarker in two different clinical scenarios: (1) mCRC patients receiving first-line chemotherapy^10^, and (2) mCRC patients receiving either anti-PD-1/PD-L1 antibodies alone or in combination with CTLA-4 blockade^11^. As already discussed by others^10,11^, one of the advantages of an integrative marker such as PIV is its capacity to rationally combine various peripheral circulating blood cells with prognostic relevance in mCRC.

Taking into account the importance of validating biomarkers in the real-world setting, in this study we investigated the utility of PIV as a prognostic biomarker in a cohort of 130 patients with CRC treated with different standard chemotherapeutic regimens in the first-line setting for metastatic disease.

Interestingly, according to the clinicopathological features of cases, we found a significantly higher median baseline PIV among those patients with ECOG-PS ≥ 2, with synchronous metastases, with ≥ 2 metastatic sites at diagnosis, with peritoneal metastases, and with bone metastases; all of them characteristics classically consider poor prognostic factors and related with disease burden. Moreover, median baseline PIV was also significantly higher among those patients who received oxaliplatin-based chemotherapy, which could be explained by an enrichment of patients with synchronous metastases in this group. Although they did not discuss these results, Corti et al.^11^ already reported in 2021 a higher median baseline PIV not only among patients with ECOG-PS ≥ 1 (vs. 0), but also among those with non-mucinous histology and with bone metastases. When considered PIV as a dichotomous variable, the proportion of patients with ECOG-PS ≥ 2, without primary tumor resection, with peritoneal metastases, and treated oxaliplatin-based chemotherapy was significantly higher in the group of patients with a baseline PIV high. In 2020, Fucà et al.^10^ reported a similar but not identical correlation, with a higher proportion of patients with ECOG-PS 1 (vs. 0), without primary tumor resection, with synchronous metastases, and with > 1 metastatic locations among patients with a baseline PIV high. Moreover, although a significantly more pronounced reduction of PIV at week 4 after therapy initiation was observed in patients without primary tumor resection, with synchronous metastases, with peritoneal metastases, and with RAS mutated tumors, no significant differences in patient and disease characteristics were observed according to early PIV increase considered as a dichotomous variable. A more pronounced early PIV variation was also described by Corti et al.^11^ among those cases with non-mucinous CRC, and among those receiving dual PD-1/CTLA-4 blockade (instead of single-agent PD-1/PD-L1 blockade). Altogether, these findings deserve further evaluation in subsequent studies.

Importantly, in our study, as previously reported by Fucà et al.^10^, a high PIV at the start of chemotherapy was associated with worse OS and PFS, which was confirmed after adjusting for various confounding factors in multivariate Cox regression analysis. However, in our study, there was not a significant correlation between baseline PIV and DCR or ORR. Importantly, we also described for the first time to the best of our knowledge, the potential utility of PIV monitoring to anticipate disease progression among those patients who achieve initial disease control with first-line chemotherapy. Among patients with no disease control (progressive disease as the best overall response), there were no significant PIV differences between week 4 and disease progression. It could be explained by the conjunction of three factors: (1) there is a significant PIV decrease at week 4 in all the cases independently of disease control status (maybe as a consequence of therapy initiation), (2) the disease is probably already progressing at week 4 in those cases with no disease control, and (3) time frame from week 4 to radiologically-documented disease progression (week 10 ± 2 weeks, or before if for medical reasons was indicated), is too short in these particular cases to detect any significant increment of PIV.

One of the potential limitations of our study is the use of only one single-centre retrospective cohort with limited sample size. Moreover, because it represents a daily clinical practice cohort, 9% of patients received a single-agent chemotherapy regimen (4% irinotecan, and 5% capecitabine/5-FU/LV). Although the effect size of the described baseline PIV correlations was congruent with the report by Fucà et al.^10^, the validation of this finding together with the role of PIV dynamics for disease monitoring (with some additional sequential determinations at different timepoints) in other independent retrospective datasets and prospective cohorts from randomized clinical trials will help to definitively confirm their clinical significance in the mCRC scenario.

In conclusion, this study besides validating baseline PIV as an independent prognostic factor for OS and PFS in mCRC, suggests the potential utility of its monitoring to anticipate the progression of the disease among those patients who achieve initial disease control with first-line standard chemotherapy. If validated in further studies, PIV may represent a useful marker for mCRC patient stratification and disease monitoring both in clinical trials and daily clinical practice.

## Patients and methods

### Study design and patient population

We conducted a single-centre retrospective study of a cohort of 130 previously untreated mCRC patients under first-line standard chemotherapy in the context of routine clinical practice between October 2015 and January 2018 from the University Clinical Hospital of Santiago de Compostela. Patients received either mFOLFOX6, FOLFIRI, or irinotecan, with or without cetuximab or bevacizumab, every 2 weeks, 5-FU/LV every 2 weeks, or capecitabine plus bevacizumab, or CAPEOX, every 3 weeks, as per standard protocol indicated, until disease progression or unacceptable toxicity.

Complete blood cell counts were extracted from electronic medical records at baseline (within a window of 30 days before the start of first-line chemotherapy), at week 4 after therapy initiation, and at documented disease progression. Demographic and clinical characteristics were also collected.

The primary efficacy endpoint was overall survival (OS). Secondary endpoints were progression-free survival (PFS), disease control rate (DCR), and overall response rate (ORR). Tumour responses were assessed by the investigators according to Response Evaluation Criteria in Solid Tumors guidelines version 1.1 every 10 ± 2 weeks or before if for medical reasons was indicated.

The study was approved by the Galician Research Ethics Committee (2015/405) and conducted in accordance with the guidelines for Good Clinical Practice and the Declaration of Helsinki. All patients provided written informed consent before enrolment.

### Statistical analysis

OS was calculated from the date of chemotherapy initiation until death resulting from any cause or last known follow-up for patients alive. PFS was calculated from the date of chemotherapy initiation until disease progression or death resulting from any cause or last known follow-up for patients with no disease progression. DCR was defined as the proportion of patients who achieved a complete or partial response and a stable disease, and ORR as the proportion of patients who achieved a complete or partial response. Patients who died before radiologic assessment were considered as not evaluable for response. PIV was calculated as [neutrophil count (10^3^/mm^3^) × platelet count (10^3^/mm^3^) × monocyte count (10^3^/mm^3^)]/lymphocyte count (10^3^/mm^3^) and dichotomized into low (≤ 390) and high (> 390) PIV as previously described^10^. Early PIV change was defined as the relative variation in PIV from baseline to week 4 after therapy initiation. Early PIV increase was defined as an early PIV change ≥  + 30%^11^. In the context of a real-world experience, Eastern Cooperative Oncology Group Performance Status (ECOG-PS) was dichotomized into two categorical variables, 0–1 versus ≥ 2. Survival estimates were calculated by the Kaplan Meier method, and groups were compared with the log-rank test. The Cox proportional hazards regression model was used to evaluate factors independently associated with OS and PFS. Baseline variables included in the multivariate analysis (forced entry method) were selected according to their clinical relevance and statistical significance in univariate analysis (cutoff, *p* < 0.05). Factors associated with DCR and ORR were tested with logistic regression in univariate analyses. Variables included in the final multivariate model were selected according to their clinical relevance and statistical significance in univariate analysis (cutoff, *p* < 0.05). Comparisons between patient and disease characteristics were carried out using Fisher’s exact test (categorical variables), and Mann–Witney U or Wilcoxon matched-pairs signed rank tests (continuous variables). All *p* values were 2-sided, and those less than 0.05 were considered statistically significant. Statistical analyses were conducted using IBM SPSS Statistics version 20.0 (IBM Corp., Armonk, NY), and GraphPad Prism version 9.2.0 (GraphPad Software, San Diego, CA).

## Supplementary Information

Supplementary Information.
