# Supplementary material for: Clinical significance of baseline Pan-Immune-Inflammation Value and its dynamics in metastatic colorectal cancer patients under first-line chemotherapy
Source: Sci Rep. 2022 Apr 27;12:6893. doi: 10.1038/s41598-022-10884-8 (PMC9046216; doi:10.1038/s41598-022-10884-8)
Supplement: Supplementary file 1 — Supplementary Information. [file 41598_2022_10884_MOESM1_ESM.pdf]

Clinical significance of baseline Pan-Immune-Inflammation Value and its dynamics in metastatic colorectal cancer patients under first-line chemotherapy

Martín Pérez-Martelo[1,2,‡], Alejandro González-García[2,‡], Yolanda Vidal-Ínsua[1,2], Cristina Blanco-Freire[1,2], Elena María Brozos-Vázquez[1,2], Ihab Abdulkader-Nallib[3], Javier Álvarez-Fernández[4], Héctor Lázare-Iglesias[3], Carolina García-Martínez[1,2], Yoel Z. Betancor[2,5], María Sánchez-Ares[3], Jose M.C. Tubío[5], Francisca Vázquez-Rivera[1,2], Sonia Candamio-Folgar[1,2,6], Rafael López-López[1,2,6,#], Juan Ruiz-Bañobre[1,2,5,6,#]

Affiliations:

[1]Medical Oncology Department, University Clinical Hospital of Santiago de Compostela, University of Santiago de Compostela (USC), 15706 Santiago de Compostela, Spain.

[2]Translational Medical Oncology Group (Oncomet), Health Research Institute of Santiago (IDIS), University Clinical Hospital of Santiago de Compostela, University of Santiago de Compostela (USC), 15706 Santiago de Compostela, Spain.

[3]Pathology Department, University Clinical Hospital of Santiago de Compostela, University of Santiago de Compostela (USC), 15706 Santiago de Compostela, Spain.

[4]Medical Oncology Department, Lucus Augusti University Hospital, 27003 Lugo, Spain.

[5]Genomes and Disease, Centre for Research in Molecular Medicine and Chronic Diseases (CiMUS), University of Santiago de Compostela (USC), 15706 Santiago de Compostela, Spain.

[6]Centro de Investigación Biomédica en Red Cáncer (CIBERONC), 28029 Madrid, Spain.

[‡]Equal contribution.

[#]Corresponding author.

Supplementary Data

- **Supplementary Tables**

  - Supplementary Table 1
  - Supplementary Table 2
  - Supplementary Table 3
  - Supplementary Table 4
  - Supplementary Table 5
  - Supplementary Table 6
  - Supplementary Table 7
  - Supplementary Table 8
  - Supplementary Table 9
  - Supplementary Table 10
  - Supplementary Table 11

Supplementary Table 1. Baseline patient and disease characteristics.

Characteristics	Patients
**Size - no.**	130
**Age - years**	
Median (range)	69 (26 - 88)
**Sex - no. (%)**	
Female	34 (26)
Male	96 (74)
**ECOG-PS - no. (%)**	
0	16 (12)
1	86 (66)
2	24 (18)
3	4 (3)
**Smoking status - no. (%)**	
Never	57 (44)
Ever	66 (51)
Current	15 (12)
Former	51 (39)
Missing	7 (5)
**Body mass index - no. (%)**	
<25	35 (27)
≥25	95 (73)
**Primary tumour site - no. (%)**	
Right colon	28 (22)
Caecum	6 (5)
Ascending colon	9 (7)
Hepatic flexure	8 (6)
Transverse colon (proximal two thirds)	5 (4)
Left colon	102 (78)
Transverse colon (distal one third)	1 (1)
Splenic flexure	3 (2)
Descending colon	6 (5)
Sigmoid colon	42 (32)
Rectosigmoid junction	17 (13)
Rectum	33 (25)
**RAS status - no. (%)**	
Mutated	68 (52)
KRAS mutation	63 (48)
NRAS mutation	5 (4)
No mutated	54 (42)
Missing	8 (6)
**BRAF status - no. (%)**	
Mutated (V600E)	2 (2)
No mutated	102 (78)
Missing	25 (19)
**MMR status - no. (%)**	
Preserve	112 (86)
Deficient	2 (2)

PMS2 deficient	1 (1)
MLH1/PMS2 deficient	1 (1)
Missing	16 (12)
**TNM stage at diagnosis - no. (%)**	
I	2 (2)
II	10 (8)
III	21 (16)
IV	97 (75)
**Site of metastases - no. (%)**	
Liver	101 (78)
Lung	64 (49)
Peritoneum	23 (18)
Lymph node	43 (33)
Bone	3 (2)
Other[a]	56 (43)
**Number of metastatic sites - no. (%)**	
1	45 (35)
2	46 (35)
3	28 (22)
4	7 (5)
5	3 (2)
6	1 (1)
**Primary tumor resection - no. (%)**	
Yes	84 (65)
R0	79 (61)
R1	1 (1)
R2	4 (3)
No	46 (35)
**Chemotherapy regimen - no. (%)**	
Oxaliplatin-based regimen	96 (74)
mFOLFOX6	89 (68)
CAPEOX	7 (5)
Non-oxaliplatin-based regimen	34 (26)
FOLFIRI	22 (17)
Irinotecan	5 (4)
Capecitabine / 5-FU/LV	7 (5)
**Previous adjuvant therapy – no. (%)**	
Adjuvant therapy	20 (15)
FOLFOX / CAPEOX	13 (10)
Capecitabine / 5-FU/LV	7 (5)
No adjuvant therapy	110 (85)
**Antibody - no. (%)**	55 (42)
Anti-EGFR	34 (26)
Cetuximab	20 (15)
Panitumumab	15 (12)
Anti-VEGF	21 (16)
Bevacizumab	21 (16)
**CEA - no. (%)**	
≤5 ng/mL	46 (35)
>5 ng/mL	84 (65)

**PIV - no. (%)**	
Low	60 (46)
High	70 (54)

Abbreviations: ECOG-PS, Eastern Cooperative Oncology Group Performance Status; CEA, carcino-embryonic antigen; PIV, Pan Immune-Inflammation Value.
[a]Ovary and fallopian tube, adrenal gland, abdominal wall and unresectable local recurrence.

Supplementary Table 2. Distribution of baseline PIV according to patient and disease characteristics.

**Characteristics**	**Total** (n = 130)	**Baseline PIV high** (n = 70, 54%)	**Baseline PIV low** (n = 60, 46%)	**p value**
**Age**				0.218
≥median	65 (50)	31 (44)	34 (57)	
<median	65 (50)	39 (56)	26 (43)	
**Sex - no. (%)**				0.552
Male	34 (26)	20 (29)	14 (23)	
Female	96 (74)	50 (71)	46 (77)	
**CEA - no. (%)**				0.017
>5 ng/mL	84 (65)	52 (74)	32 (53)	
≤5 ng/mL	46 (35)	18 (26)	28 (47)	
**BMI**				1.000
≥25	35 (27)	19 (27)	16 (27)	
<25	95 (73)	51 (73)	44 (73)	
**ECOG-PS - no. (%)**				***0.001***
≥2	28 (22)	23 (33)	5 (8)	
0-1	102 (78)	47 (67)	55 (92)	
**Chemotherapy - no. (%)**				***0.016***
Oxaliplatin-based	96 (74)	58 (83)	38 (63)	
Non-oxaliplatin-based	34 (26)	12 (17)	22 (37)	
**Synchronous metastases - no. (%)**				0.158
Yes	97 (75)	56 (80)	41 (68)	
No	33 (25)	14 (20)	19 (32)	
**Number of metastatic sites - no. (%)**				0.101
≥2	82 (63)	49 (70)	33 (55)	
1	48 (37)	21 (30)	27 (45)	
**Primary tumor location - no. (%)**				0.285
Right	28 (22)	18 (26)	10 (17)	
Left	102 (78)	52 (74)	50 (83)	
**TNM stage at diagnosis - no. (%)**				0.772
I-II	12 (9)	7 (10)	5 (8)	
III-IV	118 (91)	63 (90)	55 (92)	
**Liver metastases - no. (%)**				0.059
Yes	101 (78)	59 (84)	42 (70)	
No	29 (22)	11 (16)	18 (30)	
**Lung metastases - no. (%)**				0.603

Yes	64 (49)	36 (51)	28 (47)	
No	66 (51)	34 (49)	32 (53)	
**Peritoneal metastases - no. (%)**				***0.039***
Yes	23 (18)	17 (24)	6 (10)	
No	107 (82)	53 (76)	54 (90)	
**Lymph node metastases - no. (%)**				0.771
Yes	117 (90)	62 (89)	55 (92)	
No	13 (10)	8 (11)	5 (8)	
**Bone metastases - no. (%)**				0.249
Yes	3 (2)	3 (4)	0 (0)	
No	127 (98)	67 (96)	60 (100)	
**RAS mutation - no. (%)**				0.468
Yes	66 (54)	40 (59)	26 (48)	
No	56 (46)	28 (42)	28 (52)	
**BRAF mutation - no. (%)**				0.502
Yes	2 (2)	2 (3)	0 (0)	
No	102 (98)	56 (97)	46 (100)	
**MMR deficiency - no. (%)**				1.000
Yes	2 (2)	1 (1)	1 (2)	
No	112 (98)	60 (98)	52 (98)	
**Antibody therapy - no. (%)**				0.597
Yes	55 (42)	28 (40)	27 (45)	
No	75 (58)	42 (60)	33 (55)	
**Primary tumor resection - no. (%)**				***0.003***
Yes	84 (65)	37 (53)	47 (78)	
No	46 (35)	33 (47)	13 (22)	
**Smoking status - no. (%)**				0.224
Ever	66 (51)	32 (46)	34 (57)	
Never	64 (49)	38 (54)	26 (43)	
**Adjuvant chemotherapy - no. (%)**				0.225
Yes	20 (15)	8 (11)	12 (20)	
No	110 (85)	62 (89)	48 (80)	

Bold numbers indicate statistically significant values.

Abbreviations: ECOG-PS, Eastern Cooperative Oncology Group Performance Status; BMI, body mass index; CEA, carcino-embryonic antigen; PIV, Pan Immune-Inflammation Value.

Supplementary Table 3. Distribution of chemotherapy regimen according to patient and disease characteristics (only those statistically significant variables are shown).

**Characteristics**	**Total** (n = 130)	**Oxaliplatin-based chemotherapy** (n = 96, 74%)	**Non-oxaliplatin-based chemotherapy** (n = 34, 26%)	**p value**
**Synchronous metastases - no. (%)**				***<0.001***
Yes	97 (75)	83 (86)	14 (41)	
No	33 (25)	13 (14)	20 (59)	
**Liver metastases - no. (%)**				***0.015***
Yes	101 (78)	80 (83)	21 (62)	
No	29 (22)	16 (17)	13 (38)	
**Lymph node metastases - no. (%)**				***0.039***
Yes	117 (90)	90 (94)	27 (79)	
No	13 (10)	6 (6)	7 (21)	
**Antibody therapy - no. (%)**				***0.027***
Yes	55 (42)	35 (36)	20 (59)	
No	75 (58)	61 (64)	14 (41)	
**Primary tumor resection - no. (%)**				***0.013***
Yes	84 (65)	56 (58)	28 (82)	
No	46 (35)	40 (42)	6 (18)	
**Adjuvant chemotherapy - no. (%)**				***<0.001***
Yes	20 (15)	4 (4)	16 (47)	
No	110 (85)	92 (96)	18 (53)	

Bold numbers indicate statistically significant values.

Supplementary Table 4. Distribution of Early PIV increase according to patient and disease characteristics.

**Characteristics**	**Total** (n = 125)	**Early PIV Increase** (n = 16, 12.8%)	**No Early PIV Increase** (n = 109, 87.2%)	**p value**
**Age**				1.000
≥median	64 (51)	8 (50)	56 (51)	
<median	61 (49)	8 (50)	53 (49)	
**Sex - no. (%)**				0.554
Male	91 (73)	13 (81)	78 (72)	
Female	34 (27)	3 (19)	31 (28)	
**CEA - no. (%)**				0.416
>5 ng/mL	81 (65)	12 (75)	69 (63)	
≤5 ng/mL	44 (35)	4 (25)	40 (37)	
**BMI - no. (%)**				0.554
≥25	91 (73)	13 (81)	78 (72)	
<25	34 (27)	3 (19)	31 (28)	
**ECOG-PS - no. (%)**				0.190
≥2	27 (22)	1 (6)	26 (24)	
0-1	98 (78)	15 (94)	83 (76)	
**Chemotherapy - no. (%)**				0.370

Oxaliplatin-based	91 (73)	10 (63)	81 (74)	
Non-oxaliplatin-based	34 (27)	6 (38)	28 (26)	
**Synchronous metastases - no. (%)**				0.762
Yes	92 (74)	11 (69)	81 (74)	
No	33 (26)	5 (31)	28 (26)	
**Number of metastatic sites - no. (%)**				0.783
≥2	79 (63)	11 (69)	68 (62)	
1	46 (37)	5 (31)	41 (38)	
**Primary tumor location - no. (%)**				0.755
Right	28 (22)	4 (25)	24 (22)	
Left	97 (78)	12 (75)	85 (78)	
**TNM stage at diagnosis - no. (%)**				0.183
I-II	12 (10)	3 (19)	9 (8)	
III-IV	113 (90)	13 (81)	100 (92)	
**Liver metastases - no. (%)**				0.521
Yes	97 (78)	14 (88)	83 (76)	
No	28 (22)	2 (13)	26 (24)	
**Lung metastases - no. (%)**				0.286
Yes	60 (48)	10 (63)	50 (46)	
No	65 (52)	6 (38)	59(54)	
**Peritoneal metastases - no. (%)**				1.000
Yes	23 (18)	3 (19)	20 (18)	
No	102 (82)	13 (81)	89 (82)	
**Lymph node metastases - no. (%)**				1.000
Yes	112 (90)	15 (94)	97 (89)	
No	13 (10)	1 (6)	12 (11)	
**Bone metastases - no. (%)**				1.000
Yes	3 (2)	0 (0)	3 (3)	
No	122 (98)	16 (100)	106 (97)	
**RAS mutation - no. (%)**				0.783
Yes	63 (54)	9 (60)	54 (53)	
No	54 (46)	6 (40)	48 (47)	
**BRAF mutation - no. (%)**				1.000
Yes	2 (2)	0 (0)	2 (2)	
No	99 (98)	12 (100)	87 (98)	
**MMR deficiency - no. (%)**				1.000
Yes	2 (2)	0 (0)	2 (2)	
No	108 (98)	14 (100)	94 (98)	
**Antibody therapy - no. (%)**				0.176
Yes	55 (44)	10 (63)	45 (41)	
No	70 (56)	6 (38)	64 (59)	
**Primary tumor resection - no. (%)**				0.415
Yes	81 (65)	12 (75)	69 (63)	
No	44 (35)	4 (25)	40 (37)	
**Smoking status - no. (%)**				0.791
Ever	61 (49)	7 (44)	54 (50)	

Never	64 (51)	9 (56)	55 (50)	
**Adjuvant chemotherapy - no. (%)**				1.000
Yes	20 (16)	2 (13)	18 (17)	
No	105 (84)	14 (87)	91 (83)	

Abbreviations: ECOG-PS, Eastern Cooperative Oncology Group Performance Status; BMI, body mass index; CEA, carcino-embryonic antigen; PIV, Pan Immune-Inflammation Value.

Supplementary Table 5. Efficacy endpoints.

**Endpoints**	**Results**
**Response - no. (%)**	
Complete response	1 (0.77)
Partial response	68 (52.31)
Stable disease	33 (25.38)
Progressive disease	17 (13.08)
Not evaluable	11 (8.46)
**Overall response rate - % (95% CI)**	53.08 (41.30 - 67.17)
**Disease control rate - % (95% CI)**	78.46 (63.98 - 95.25)
**Median overall survival - months (95% CI)**	21.14 (17.70 - 24.78)
**6-month overall survival rate - % (95% CI)**	74.62 (60.51 - 91.02)
**12-month overall survival rate - % (95% CI)**	65.38 (52.23 - 80.85)
**24-month overall survival rate - % (95% CI)**	42.31 (31.87 - 55.07)
**36-month overall survival rate - % (95% CI)**	22.31 (14.94 - 32.04)
**Median progression-free survival - months (95% CI)**	9.30 (7.62 - 10.98)

Abbreviations: CI, confidence interval.

Supplementary Table 6. Univariate and multivariate Cox regression analyses for overall survival (combination chemotherapy subgroup).

	**Univariate analysis**		**Multivariate analysis**	
**Characteristics**	**HR (95% CI)**	**p value**	**HR (95% CI)**	**p value**
Age (increment of one year)	1.018 (0.997 - 1.04)	0.077		
Sex (male vs female)	1.135 (0.695 - 1.854)	0.61		
CEA (>5 ng/mL vs ≤5 ng/mL)	2.366 (1.468 - 3.814)	***0.0004***	1.917 (1.109 - 3.314)	***0.02***
BMI (≥25 vs <25)	0.782 (0.491 - 1.245)	0.3		
ECOG-PS (≥2 vs 0-1)	3.349 (2.026 - 5.535)	***0.000002***	2.591 (1.409 - 4.765)	***0.002***
Baseline PIV (high vs low)	2.287 (1.478 - 3.54)	***0.0002***	1.839 (1.125 - 3.008)	***0.015***
Chemotherapy (oxaliplatin-based vs non-oxaliplatin-based)	1.424 (0.817 - 2.483)	0.212		
Synchronous metastases (yes vs no)	1.439 (0.874 - 2.37)	0.152		
Number of metastatic sites (≥2 vs 1)	2.47 (1.555 - 3.924)	***0.0001***	1.254 (0.673 - 2.335)	0.476
Primary tumor location (right vs left)	1.604 (0.979 - 2.629)	0.06		
TNM stage at diagnosis (III-IV vs I-II)	1.328 (0.641 - 2.753)	0.444		
Liver metastases (yes vs no)	1.433 (0.861 - 2.384)	0.165		

Lung metastases (yes vs no)	1.933 (1.265 - 2.953)	***0.002***	1.559 (0.843 - 2.88)	0.157
Peritoneal metastases (yes vs no)	1.558 (0.915 - 2.655)	0.102		
Lymph node metastases (yes vs no)	0.491 (0.252 - 0.958)	***0.036***	0.395 (0.191 - 0.818)	***0.012***
Bone metastases (yes vs no)	6.129 (1.458 - 25.752)	***0.013***	2.05 (0.436 - 9.636)	0.363
RAS mutation (yes vs no)	1.112 (0.722 - 1.711)	0.629		
BRAF mutation (yes vs no)	2.495 (0.600 - 10.368)	0.208		
MMR deficiency (yes vs no)	3.402 (0.812 - 14.247)	0.094		
Antibody therapy (yes vs no)	0.6 (0.389 - 0.928)	***0.021***	0.626 (0.9 - 1.004)	0.052
Primary tumor resection (yes vs no)	0.33 (0.214 - 0.507)	***0.0000004***	0.445 (0.268 - 0.738)	***0.002***
Smoking status (ever vs never)	1.004 (0.662 - 1.522)	0.984		
Adjuvant chemotherapy (yes vs no)	0.659 (0.358 - 1.214)	0.181		

Bold numbers indicate statistically significant values.
Abbreviations: HR, hazard ratio; CI, confidence interval; ECOG-PS, Eastern Cooperative Oncology Group Performance Status; BMI, body mass index; CEA, carcino-embryonic antigen; PIV, Pan Immune-Inflammation Value.

Supplementary Table 7. Univariate and multivariate Cox regression analyses for progression-free survival (combination chemotherapy subgroup).

	Univariate analysis		Multivariate analysis	
**Characteristics**	**HR (95% CI)**	**p value**	**HR (95% CI)**	**p value**
Age (increment of one year)	1.013 (0.994 - 1.033)	0.175		
Sex (male vs female)	0.883 (0.573 - 1.361)	0.574		
CEA (>5 ng/mL vs ≤5 ng/mL)	1.756 (1.165 - 2.648)	***0.007***	1.34 (0.818 - 2.196)	0.245
BMI (≥25 vs <25)	0.588 (0.385 - 0.896)	***0.014***	0.485 (0.302 - 0.779)	***0.003***
ECOG-PS (≥2 vs 0-1)	2.756 (1.695 - 4.482)	***0.00004***	1.871 (1.042 - 3.356)	***0.036***
Baseline PIV (high vs low))	2.112 (1.417 - 3.147)	***0.00024***	1.517 (0.98 - 2.35)	0.062
Chemotherapy (oxaliplatin-based vs non-oxaliplatin-based)	1.318 (0.81 - 2.147)			
Synchronous metastases (yes vs no)	1.653 (1.03 - 2.652)	***0.037***	0.869 (0.489 - 1.545)	0.632
Number of metastatic sites (≥2 vs 1)	2.393 (1.588 - 3.606)	***0.00003***	1.844 (1.04 - 3.268)	***0.036***
Primary tumor location (right vs left)	1.41 (0.884 - 2.248)	0.149		
TNM stage at diagnosis (III-IV vs I-II)	1.624 (0.789 - 3.346)	0.188		
Liver metastases (yes vs no)	1.324 (0.839 - 2.091)	0.228		
Lung metastases (yes vs no)	2.186 (1.477 - 3.236)	***0.0001***	1.481 (0.86 - 2.552)	0.1571
Peritoneal metastases (yes vs no)	1.259 (0.754 - 2.102)	0.378		
Lymph node metastases (yes vs no)	0.662 (0.343 - 1.278)	0.219		
Bone metastases (yes vs no)	5.637 (1.343 - 23.654)	***0.018***	3.365 (0.74 - 15.313)	0.116
RAS mutation (yes vs no)	0.872 (0.586 - 1.296)	0.499		
BRAF mutation (yes vs no)	2.637 (0.631 - 11.007)	0.183		
MMR deficiency (yes vs no)	1.887 (0.461 - 7.737)	0.377		
Antibody therapy (yes vs no)	0.656 (0.441 - 0.975)	***0.037***	0.668 (0.427 - 1.043)	0.076
Primary tumor resection (yes vs no)	0.353 (0.234 - 0.531)	***0.000001***	0.516 (0.313 - 0.848)	***0.009***
Smoking status (ever vs never)	0.785 (0.535 - 1.152)	0.216		
Adjuvant chemotherapy (yes vs no)	0.601 (0.342 - 1.056)	0.077		

Supplementary Table 8. Univariate and multivariate logistic regression analysis for disease control rate.

	Univariate analysis		Multivariate analysis	
**Characteristics**	**OR (95% CI)**	**p value**	**OR (95% CI)**	**p value**
Age (increment of one year)	0.984 (0.945 - 1.025)	0.437		
Sex (male vs female)	2.223 (0.913 - 5.408)	0.078		
ECOG-PS (≥2 vs 0-1)	0.483 (0.189 - 1.233)	0.128		
Smoking status (ever vs never)	1.809 (0.771 - 4.242)	0.173		
CEA (>5 ng/mL vs ≤5 ng/mL)	0.423 (0158 - 1.133)	***0.087***	0.702 (0.214- 2.304)	0.560
Baseline PIV (high vs low)	0.700 (0.299 - 1.641)	0.412		
BMI (≥25 vs <25)	1.711 (0.699 - 4.188)	0.240		
Primary tumor location (right vs left)	1.339 (0.458 - 3.915)	0.594		
TNM stage at diagnosis (III-IV vs I-II)	0.708 (0.146 - 3.434)	0.668		
Synchronous metastases (yes vs no)	0.420 (0.134 - 1.315)	0.136		
Primary tumor resection (yes vs no)	3.890 (1.627 - 9.304)	***0.002***	2.006 (0.706- 5.701)	0.191
Chemotherapy (oxaliplatin-based vs non-oxaliplatin-based)	0.400 (0.128 - 1.252)	0.115		
Adjuvant chemotherapy (yes vs no)	6.181 (0.790 - 48.360)	0.083		
Antibody therapy (yes vs no)	6.000 (1.943 - 18.526)	***0.002***	5.583 (1.728 - 18.039)	***0.004***
Number of metastatic sites (≥2 vs 1)	0.390 (0.145 - 1.043)	0.061		
Liver metastases (yes vs no)	0.214 (0.047 - 0.961)	***0.044***	0.278 (0.053 – 1.473)	0.132
Lung metastases (yes vs no)	0.374 (0.154 - 0.905)	***0.029***	0.611 (0.219 – 1.705)	0.347
Peritoneal metastases (yes vs no)	1.373 (0.426 - 4.425)	0.595		
Lymph node metastases (yes vs no)	1.104 (0.282 - 4.318)	0.887		
Bone metastases (yes vs no)	0.540 (0.047 - 6.182)	0.620		
RAS mutation (yes vs no)	0.522 (0.206 - 1.322)	0.170		
BRAF mutation (yes vs no)	394018254.354	0.999		
MMR deficiency (yes vs no)	394893850.475	0.999		

Bold numbers indicate statistically significant values.
Abbreviations: OR, odds ratio; CI, confidence interval; ECOG-PS, Eastern Cooperative Oncology Group Performance Status; BMI, body mass index; CEA, carcino-embryonic antigen; PIV, Pan Immune-Inflammation Value.

Supplementary Table 9. Univariate and multivariate logistic regression analysis for overall response rate.

	Univariate analysis		Multivariate analysis	
**Characteristics**	**OR (95% CI)**	**p value**	**OR (95% CI)**	**p value**
Age (increment of one year)	1.012 (0.980 - 1.044)	0.463		
Sex (male vs female)	1.182 (0.540 - 2.586)	0.676		
ECOG-PS (≥2 vs 0-1)	0.712 (0.308 - 1.647)	0.427		
Smoking status (ever vs never)	0.996 (0.500 - 1.984)	0.991		
CEA (>5 ng/mL vs ≤5 ng/mL)	1.819 (0.880 - 3.764)	0.106		
Baseline PIV (high vs low)	0.981 (0.492 - 1.958)	0.957		
BMI (≥25 vs <25)	1.280 (0.589 - 2.783)	0.533		
Primary tumor location (right vs left)	1.233 (0.530 - 2.865)	0.627		
TNM stage at diagnosis (III-IV vs I-II)	0.200 (0.042 - 0.952)	***0.043***	0.178 (0.036 - 0.880)	***0.034***
Synchronous metastases (yes vs no)	1.088 (0.493 - 2.398)	0.835		
Primary tumor resection (yes vs no)	1.386 (0.674 - 2.851)	0.375		
Chemotherapy (oxaliplatin-based vs non-oxaliplatin-based)	1.182 (0.540 - 2.586)	0.676		
Adjuvant chemotherapy (yes vs no)	0.682 (0.262 - 1.776)	0.433		
Antibody therapy (yes vs no)	3.173 (1.523 - 6.608)	***0.002***	3.336 (1.576 - 7.062)	***0.002***
Number of metastatic sites (≥2 vs 1)	1.587 (0.775 - 3.250)	0.207		
Liver metastases (yes vs no)	1.835 (0.795 - 4.239)	0.155		
Lung metastases (yes vs no)	0.784 (0.393 - 1.563)	0.489		
Peritoneal metastases (yes vs no)	0.774 (0.314 - 1.909)	0.579		
Lymph node metastases (yes vs no)	1.361 (0.431 - 4.296)	0.599		
Bone metastases (yes vs no)	1.791 (0.158 - 20.255)	0.638		
RAS mutation (yes vs no)	0.600 (0.291 - 1.239)	0.167		
BRAF mutation (yes vs no)	0.729 (0.044 - 11.979)	0.825		
MMR deficiency (yes vs no)	1451189265.612	0.999		

Bold numbers indicate statistically significant values.
Abbreviations: OR, odds ratio; CI, confidence interval; ECOG-PS, Eastern Cooperative Oncology Group Performance Status; BMI, body mass index; CEA, carcino-embryonic antigen; PIV, Pan Immune-Inflammation Value.

Supplementary Table 10. Univariate Cox regression analysis for survival.

Characteristics	HR (95% CI)	p value
**Overall survival**		
Early PIV increase (yes vs no)	1.403 (0.796 - 2.474)	0.242
**Progression-free survival**		
Early PIV increase (yes vs no)	1.359 (0.787 - 2.345)	0.271

Abbreviations: CI, confidence interval; HR, hazard ratio; PIV, Pan Immune-Inflammation Value.

Supplementary Table 11. Logistic regression analysis for disease control and overall response rates.

Characteristics	OR (95% CI)	p value
**Disease control rate**		
Early PIV increase (yes vs no)	0.802 (0.237 - 2.722)	0.724
**Overall response rate**		
Early PIV increase (yes vs no)	1.520 (0.517 - 4.476)	0.447

Abbreviations: CI, confidence interval; HR, hazard ratio; PIV, Pan Immune-Inflammation Value.
